# Inhibition of CREB binding protein-beta-catenin signaling down regulates CD133 expression and activates PP2A-PTEN signaling in tumor initiating liver cancer cells

**DOI:** 10.1186/s12964-018-0222-5

**Published:** 2018-03-12

**Authors:** Yuanyuan Tang, Joshua Berlind, Nirmala Mavila

**Affiliations:** 1 Department of Oncology, The Second Xiangya Hospital, Central South University, Changsha, Hunan 410011 China; 2 Division of Digestive and Liver Diseases, Department of Medicine, Cedars-Sinai Medical Center, Los Angeles, CA 90048 USA; 3 Division of Applied Cell Biology and Physiology, Department of Biomedical Sciences, Cedars-Sinai Medical Center, Los Angeles, CA 90048 USA

**Keywords:** WNT-beta catenin signaling, Tumor initiating cells, Cancer stem cells, CBP, PTEN, CD133, PP2A

## Abstract

**Background:**

The WNT-beta-catenin pathway is known to regulate cellular homeostasis during development and tissue regeneration. Activation of WNT signaling increases the stability of cytoplasmic beta-catenin and enhances its nuclear translocation. Nuclear beta-catenin function is regulated by transcriptional co-factors such as CREB binding protein (CBP) and p300. Hyper-activated WNT-beta-catenin signaling is associated with many cancers. However, its role in inducing stemness to liver cancer cells, its autoregulation and how it regulates tumor suppressor pathways are not well understood. Here we have investigated the role of CBP-beta-catenin signaling on the expression of CD133, a known stem cell antigen and PP2A-PTEN pathway in tumor initiating liver cancer cells.

**Methods:**

Human hepatoblastoma cell line HepG2 and clonally expanded CD133 expressing tumor initiating liver cells (TICs) from premalignant murine liver were used in this study. CBP-beta-catenin inhibitor ICG001 was used to target CBP-beta catenin signaling in liver cancer cells in vitro*.* Western blotting and real time PCR (qPCR) were used to quantify protein expression/phosphorylation and mRNA levels, respectively. *CBP* and *CD133* gene silencing was performed by siRNA transfection. Fluorescence Activated Cell Sorting (FACS) was performed to quantify CD133 positive cells. Protein Phosphatase (PP2A) activity was measured after PP2AC immunoprecipitation.

**Results:**

CBP inhibitor ICG001 and *CBP* silencing significantly reduced *CD133* expression and anchorage independent growth in HepG2 and murine TICs. *CD133* silencing in TICs decreased cell proliferation and expression levels of cell cycle regulatory genes, *CyclinD1* and *CyclinA2*. ICG001 treatment and *CBP* silencing reduced the levels of phospho^Ser380/Tyr382/383^PTEN, phospho^Ser473^-AKT, Phospho-^Ser552^beta-catenin in TICs. ICG001 mediated de-phosphorylation of PTEN in TICs was PP2A dependent and partly prevented by co-treatment with PP2A inhibitor okadaic acid.

**Conclusions:**

CBP-beta-catenin signaling promotes stemness via CD133 induction and cell proliferation in TICs. We found a novel functional link between CBP-beta-catenin and PP2A-PTEN-AKT pathway in liver TICs. Therefore, CBP-beta-catenin-PP2A-PTEN-AKT signaling axis could be a novel therapeutic target to prevent liver tumor initiation and cancer recurrence.

**Electronic supplementary material:**

The online version of this article (10.1186/s12964-018-0222-5) contains supplementary material, which is available to authorized users.

## Background

WNT-beta-catenin signaling is one of the most conserved signaling pathways that is critical for the development and regeneration of various organs. It is highly deregulated in many cancers. Beta-catenin is the central component of the canonical WNT pathway. Secreted WNT ligands interact with Frizzled/LRP (low density lipoprotein receptor-related protein) receptors to activate intracellular WNT signaling [1, 2]. Cytosolic beta-catenin stability and its nuclear translocation is regulated by beta-catenin degradation complex, which consists of Glycogen Synthase Kinase-3-beta, Axin, Adenomatous polyposis coli (APC) and Casein kinase1 (CK1). When WNT signaling is activated, the beta-catenin degradation complex gets inactivated and beta-catenin accumulates in the cytoplasm and translocates to the nucleus where it binds to the T-Cell Factor /Lymphoid Enhancer-Binding Factor (TCF/LEF) family of transcription factors and activates the expression of WNT target genes [1]. Tightly regulated dynamic protein modifications such as phosphorylation play an important role in the formation and function of the beta-catenin degradation complex. In the nucleus, beta-catenin activity is further regulated by its interaction with transcriptional co-activators. Two such nuclear transcriptional co-activators are CREB-binding protein (CBP) and its homolog p300. Beta-catenin directly interacts with CBP to promote survival and proliferation of embryonic and somatic stem cells, whereas its interaction with p300 promotes differentiation of embryonic stem cells [3].

Recent studies have shown that cancer recurrence is associated with the presence of tumor initiating cells (TICs) [4, 5]. TICs are a small population of cells that have high proliferative capacity and self-renewal with characteristics of drug resistance [6]. TICs are characterized by the expression of various stem cell markers such as CD133 (PROM1), CD24, CD44, EpCAM and LGR5 [7, 8]. CD133 is a microvillus specific membrane glycoprotein believed to maintain stemness of cells [9, 10]. Rountree et al. successfully isolated CD133 expressing cells from premalignant methionine adenosyltransferase1-alpha knock-out murine livers and developed clonally expanded tumor initiating cell line (TICs) [11]. These CD133 enriched TICs form tumors in xenograft models compared to CD133 negative liver cells [12]. CD133 positive cells have had increased survival in vitro and have been shown to have drug resistance in vivo [13, 14]. Recent studies increasingly support the concept that activated WNT signaling is associated with cancer stem cells, however the molecular mechanism how this signaling pathway induces stemness to cancer cells and promotes self-renewal is still an area of active investigation [15–18].

ICG001 is a small molecule inhibitor that disrupts CBP-beta-catenin signaling [19]. ICG001 has been shown to reduce tumor growth both in vitro and in vivo xenograft models [20]. Using ICG001, we previously have demonstrated that CBP-beta-catenin signaling regulates the survival and proliferation of CD133 enriched murine embryonic liver cells and clonally expanded CD133 positive TICs [21]. Here we extended our efforts to elucidate the functional role of CBP-beta-catenin signaling in inducing stemness to human liver cancer cells and murine TICs. Previously we have also shown that AKT positively regulates CBP-beta-catenin signaling and promotes proliferation of TICs [21]. Since we found that CBP-beta-catenin signaling is highly activated in TICs and required for its survival and proliferation, we extended our efforts to identify the mechanism of self-regulation of CBP-beta-catenin pathway in TICs. Our results show that CBP-beta-catenin signaling induce stemness in liver TICs via CD133 expression and is self-regulated by suppressing PP2A-PTEN and downstream activation of AKT. Therefore, targeting CBP-beta-catenin-PP2A-PTEN-AKT crosstalk may hold a novel therapeutic approach to specifically target tumor initiating liver cancer cells.

## Methods

All general buffers and chemicals were purchased from Sigma-Aldrich and were of molecular biology grade.

### Cell culture

Clonally expanded murine CD133 positive (TICs) cells (kindly provided by Dr. Bart Rountree) originally described by Rountree et al. [11] was cultured on standard tissue culture plates (BD Biosciences) in DMEM (Invitrogen) containing 10% Fetal Bovine Serum (FBS, Invitrogen), 100 units/100 μg/ml Penicillin/Streptomycin (Gibco, Carlsbad, CA), 1 μg/ml Insulin (Sigma-Aldrich, St. Louis, MO), 10^− 7^ M dexamethasone (Sigma-Aldrich), and 10 mM nicotinamide (Sigma-Aldrich) in a cell culture incubator maintained at 37 °C and 5% CO_2_. Human hepatocellular carcinoma cell line HepG2 and cholangiocarcinoma cell line MzCha-1 (kindly provided by Dr. Shelly Lu, Cedars Sinai Medical Center) were grown as described previously [22]. Cells were trypsinized with 0.05% Trypsin-EDTA (Gibco, Carlsbard, CA) and passaged every 3 days. For serum starvation, cells were washed in sterile phosphate buffered saline (PBS) twice and media was changed to DMEM containing dexamethasone and nicotinamide minus serum and insulin for 16 h. For CBP loss-of-function experiments, we utilized ICG001 (Selleckchem, Houston, TX), a small molecule inhibitor that specifically disrupts beta-catenin interaction with CBP at an established dose of 10 μM concentration as described previously [19, 21, 23].

### Fluorescence activated cell sorting (FACS) analysis

FACS analysis was performed as described previously [24]. HepG2 cells or TICs were treated with ICG001 for 48 h as described before. One million live cells were Fc blocked, incubated with two microgram of anti-PROM1-Phycoerythrin (Miltenyi Biotech, Auburn, CA (human)/ eBiosciences (mouse), San Diego, CA), and washed with ice-cold PBS prior to analysis on a BD LSRII Flow Cytometer (San Jose, CA). Gating was determined by unstained cells. DAPI staining was performed to gate live cells. Isotype IgG-stained controls were used to exclude non-specific stained cells.

### In vitro gene silencing

Pre-validated Silencer® select siRNAs against *CBP, CD133* and control scrambled siRNA were purchased from Thermo Scientific (Rockford, IL). The siRNA was reverse transfected into 1 × 10^5^ cells at a dose of 20 nM in 6-well plates using the Lipofectamine RNAiMAX™ transfection reagent (Invitrogen, Carlsbad, CA) for 48 h as described previously [22].

### Anchorage-independent growth assay

Anchorage-independent growth assay was performed as described [25]. Briefly, HepG2 cells or TICs (1.5 × 10^3^ per well) were grown in 0.7% top soft agar prepared on a 0.5% base soft agar layer in a 6-well plate for two weeks in Dulbecco’s modified Eagle medium supplemented with 10% fetal bovine serum with and without ICG001. Colonies formed at the end of two weeks were stained with 0.005% crystal violet for 30 min and washed thoroughly with water, and images were acquired using an Evos Advanced transmitted light microscope coupled with Evos *xl* software (AMG, Bothell, WA). Number of colonies was counted manually from five different images captured from six independent experiments.

### Immunocytochemistry

Immunocytochemistry was performed as described previously [21]. Briefly, after treatment, cells were fixed in 4% Para-formaldehyde for 30 min at room temperature and washed in PBS (Phosphate buffer-saline) twice for 5 min. Then the cells were permeabilized with Tris-buffered saline-Triton X-100 (0.5%) for 10 min and then washed in PBS for five minutes twice. Nonspecific antibody binding was blocked by incubating with 5% goat serum (Sigma-Aldrich) in TBST (Tris buffer saline-tween-20, 0.1%) for 45 min at room temperature. Cells were incubated with primary antibody diluted in 5% goat serum for 16 h at 4 °C. Signals were detected by secondary antibody conjugated with goat-anti-mouse Cy3 (1:200, Jackson Immuno Research Laboratories, West Grove, PA, Abcam). Fluorescence images were acquired with KEYENCE al BZ-X710 inverted fluorescent microscope (KEYENCE Corporation of America, Itasca, IL, USA).

### Western blot analysis

Total protein lysates were prepared from cells using Radio Immuno Precipitation Assay (RIPA) buffer (50 mM Tris-HCl, pH 7.5, 150 mM NaCl, 0.1% Triton X-100, 0.1% Sodium deoxycholate, 1 mM EDTA, 1 mM Phenyl methyl sulphonyl fluoride (Sigma-Aldrich), Phosphatase inhibitor cocktail (Thermo Scientific) and protease inhibitor cocktail (Sigma-Aldrich). Protein concentrations were measured by Bradford’s protein assay kit (Bio-Rad Laboratories) using bovine serum albumin as standard. Equal amounts of protein samples were separated on a 10% SDS-PAGE at 100 V and transferred onto nitrocellulose membrane (Bio-Rad). After blocking with 5% BSA or BLOTTO (Santa Cruz Biotechnology) prepared in Tris-buffered saline, Tween, 0.1%, (TBST), membranes were incubated with respective primary antibody diluted in blocking buffer for 16 h at 4 °C. Membranes were then washed in TBST and incubated with horseradish peroxidase-conjugated secondary antibody. Primary antibodies used are listed in Additional file 1: Table S1. Finally, signals were detected using Millipore chemi-luminescence western blot detection reagent. ImageJ software (NIH) was used to measure the protein band intensity. Beta-actin was used as loading control. Phosphorylated proteins were normalized to its non-phosphorylated form to determine the fold activation.

### RNA isolation, reverse transcription, and quantitative real-time PCR

DNA-free RNA was isolated using a column-based purification method according to the manufacturer’s protocol (Quick- RNA TM Miniprep, Zymo Research, Irvine, CA). One microgram of total RNA was reverse-transcribed using 100 units of NxGen® M-MuLV Reverse Transcriptase according to the manufacturer’s protocol (Lucigen Corp., Middleton, WI). Quantitative real-time PCR (qPCR) was performed using gene specific Taq-Man probes (Additional file 2: Table S2) described previously [22]. Relative mRNA levels were calculated from cycle threshold (Ct Value) by Δ–ΔC_t_. *Gapdh* was used to normalize the gene expression.

### PP2A activity

PP2A activity was measured using PP2A Immunoprecipitation-Phosphatase Assay Kit from EMD Millipore (catalog#17–313, Billerica, MA) according to manufacturer’s protocol. Briefly, 1 × 10^6^ cells were synchronized by serum starvation for 16 h and performed ICG001 treatment for 24 h. Cells were lysed by sonication in ice-cold imidazole buffer containing protease inhibitor cocktail (Sigma-Aldrich). Samples were spun at 10,000×g and clear supernatant was used for immunoprecipitation. Each sample (500 microgram total protein) was subjected to immunoprecipitation with PP2Ac antibody for 16 h. Immuno-beads with PP2A were added to a phosphatase reaction mix containing threonine phospho-peptide in a shaking incubator for 15 min. Samples were then spun down and supernatant was transferred into a 96-well plate, into which malachite green detection solution was added. Plates were incubated for 15 min at room temperature and then read the absorbance at 650 nm in an automated plate reader (Molecular Devices, Sunnyvale, CA).

### Statistical analysis

All data shown represent the mean ± SEM. The statistical significance of differences between two groups was analyzed with two-sided unpaired Student’s *t* tests. Statistical significance was defined by *P* < 0.05.

## Results

### ICG001 down regulates CD133 expression and anchorage independent growth in murine TICs

**Fig. 1:**
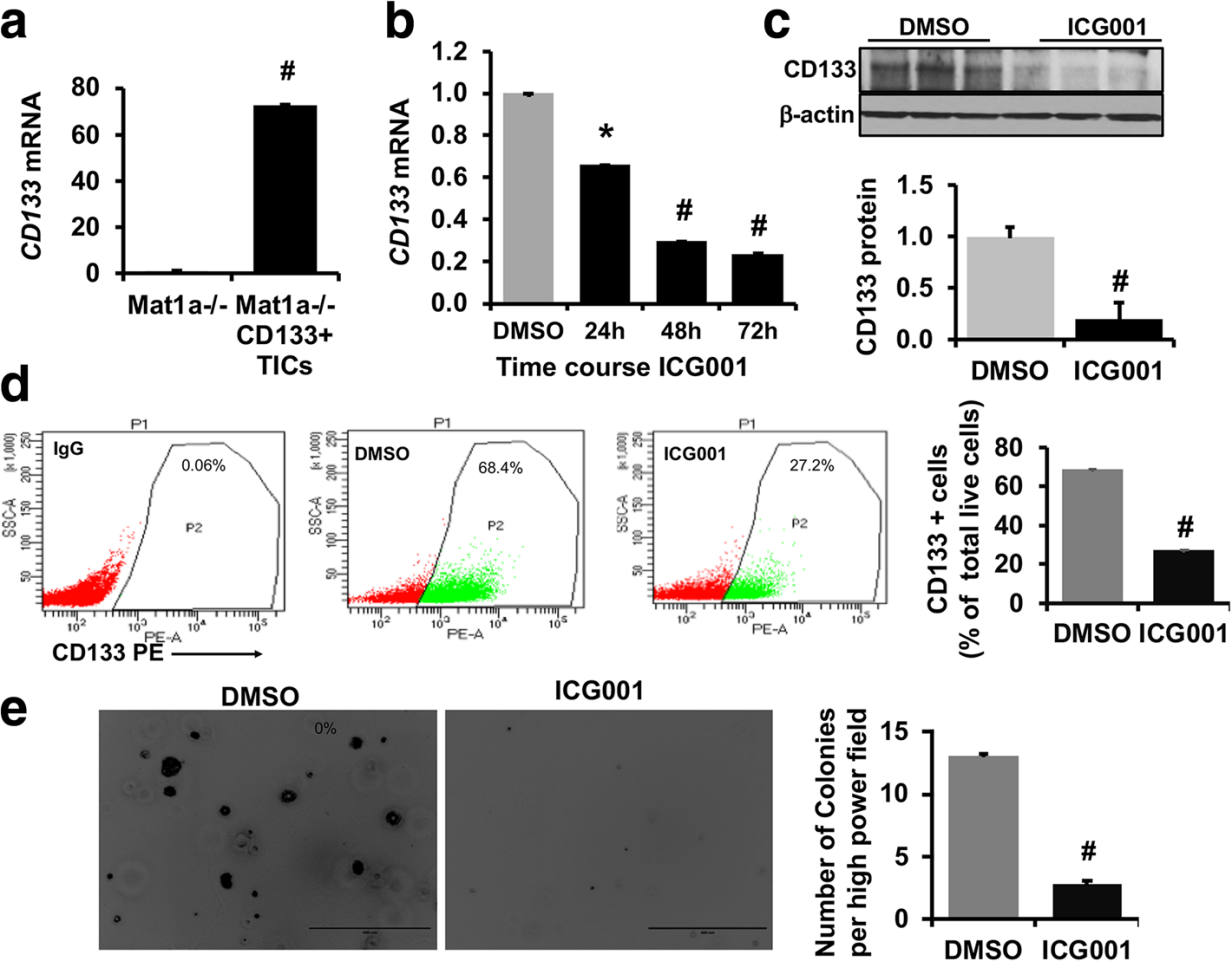
CBP-beta-catenin signaling regulates CD133 expression and anchorage independent growth in murine TICs: **a** Expression levels of *CD133* mRNA in *CD133 positive* clonally expanded TICs from *Mat1a−/−* murine liver and liver cancer cell line developed from *Mat1a*−/− mouse liver tumors. Data represents mean ±S.E from three independent experiments (*n* = 3, #*p* < 0.0001). **b** Effect of time course ICG001 treatment on *CD133* expression in TICs. Results represent mean ±S.E from four independent experiments (*n* = 4, *p < 0.005, # *p* < 0.0005) **c** Western blot to show the effect of ICG001 on CD133 protein levels. Protein band intensity was calculated by densitometry quantification by Image J Software, NIH. Data is represented as fold difference compared to DMSO treated control cells. Data represents mean ±S.E from three independent experiments (*n* = 3, #*p* < 0.005). **d** FACS analysis to show the effect of ICG001 on CD133 positive cells in TICs. Data represented as % of total live cells in each group. Live cells were gated with DAPI staining. Results represent mean ±S.E from three independent experiments (*n* = 3, # *p* < 0.001). **e** Effect of ICG001 on anchorage independent growth in TICs. After 14 days of culture on soft agar with and without ICG001, colonies were stained with 0.005% crystal violet and images were captured. For each experiment 4–5 images were captured from six different experiments and colonies were counted manually and average number of colonies were shown in the graph. *n* = 6, # *p* <  0.005

We previously have reported that ICG001 mediated inhibition of CBP-beta-catenin signaling down regulates proliferation of clonally expanded murine TICs [21]. TICs expressed ~ 70-fold higher *CD133* mRNA levels compared to *Mat1a−/−* murine liver tumor cells (Fig. 1a). To determine whether CBP-beta-catenin signaling regulates *CD133* expression, time course ICG001 treatment was performed in TICs. ICG001 treatment resulted in a time dependent decrease in *CD133* mRNA expression with ~ 80% drop by 72 h of treatment (Fig. 1b). Western blot showed about 80% decrease in CD133 protein levels and FACS analysis showed a 2.5- fold decrease in CD133 positive cells in ICG001 treated cells (Fig. 1c-d). To determine whether ICG001 treatment affects in vitro tumorigenicity of TICs, soft agar colony formation agar assay was performed. The number of colonies was significantly reduced in ICG001 treated TICs compared to DMSO treated control cells (Fig. 1e).

### ICG001 down regulates CD133 expression, cell survival genes and abrogates anchorage independent growth in HepG2 cells

**Fig. 2:**
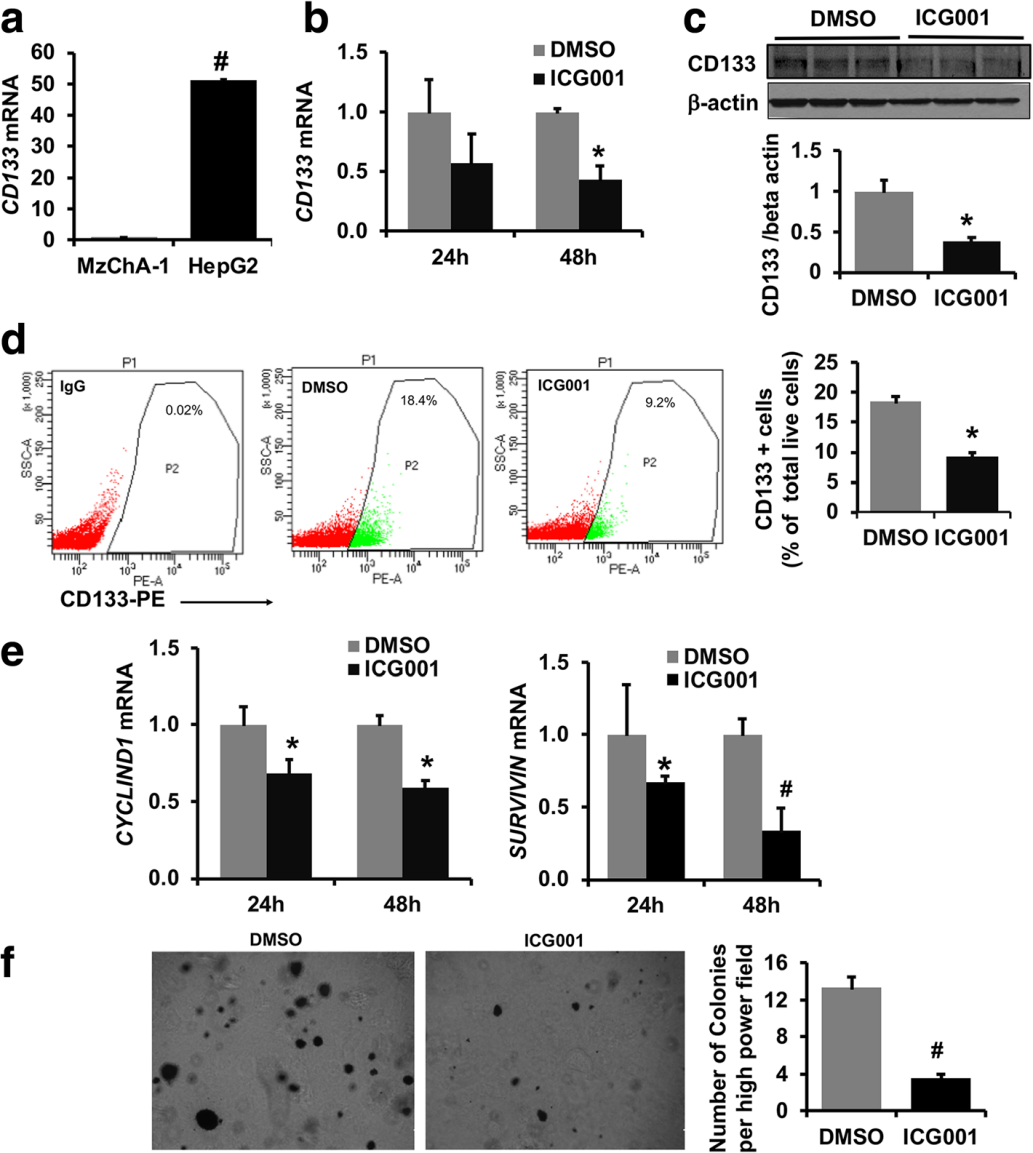
CBP-beta-catenin signaling regulates CD133 expression, cell survival proliferative genes and anchorage independent growth in HepG2 cells. **a ***CD133* mRNA expression levels in human hepatoblastoma cell line HepG2 and cholangiocarcinoma cell line MzCha-1. Expression level was normalized to MzCha-1 cell line and fold expression was calculated, (*n* = 3 #*p* < 0.005). **b** Effect of time course ICG001 treatment on *CD133* mRNA expression (*n* = 3 *p < 0.001). **c** Western blot to show the effect of ICG001 on CD133 protein levels. Protein band intensity was calculated by densitometry quantification by Image J Software, NIH. Results represent mean ±S.E from three independent experiments (*n* = 3, # *p* < 0.01). **d** FACS analysis to show the effect of ICG001 on CD133 positive cells in HepG2 cells. Data represented as % of total live cells in each group. Live cells were gated with DAPI staining. Results represent mean ±S.E from three independent experiments (*n* = 3, # *p* < 0.001). **e** Effect of ICG001 on *CYCLIND1* and *SURVIVIN* expression levels in HepG2 cells. Results represent mean ±S.E from three independent experiments #*p* < 0.001, *p < 0.05. **f** Effect of ICG001 on anchorage independent growth in HepG2 cells. After 14 days of culture on soft agar with and without ICG001, colonies were stained with 0.005% crystal violet and images were captured. For each experiment 4–5 images were captured from six different experiments and colonies were counted manually and average number of colonies were shown in the graph, *n* = 6, # *p* < 0.005

Next, we investigated the expression levels of *CD133* in human liver cancer cell lines. qPCR demonstrated that the human HCC cell line HepG2 cells expressed ~ 50-fold high levels of *CD133* compared to CCA cell line, MzChA-1 cells (Fig. 2a). To examine whether *CD133* expression is regulated by CBP-beta-catenin, HepG2 cells were treated with ICG001 for 24 h and 48 h. ICG001 treatment resulted in a time dependent decrease in *CD133* mRNA expression with ~ 70% decrease at 48 h compared to DMSO treated cells (Fig. 2b). ICG001 treatment for 48 h decreased CD133 protein expression by 63% (Fig. 2c). FACS analysis further demonstrated that ICG001 treatment reduced the number of CD133 positive cells by 2-fold after 48 h (Fig. 2d). qPCR was performed to validate ICG001 activity and found that known CBP targets, *CYCLIND1* and *SURVIVIN* levels were significantly reduced by 42% and 67% respectively after 48 h of treatment (Fig. 2e). Next, we analyzed the ability of HepG2 cells to propagate and form colonies on soft agar in the presence of ICG001. Figure 2f shows that ICG001 abrogates anchorage independent growth of HepG2 cells on soft agar and a ~ 3.5-fold decrease in number of colonies was observed in ICG001 treated cells compared to DMSO controls.

### CD133 expression is associated with cell proliferation in TICs

**Fig. 3:**
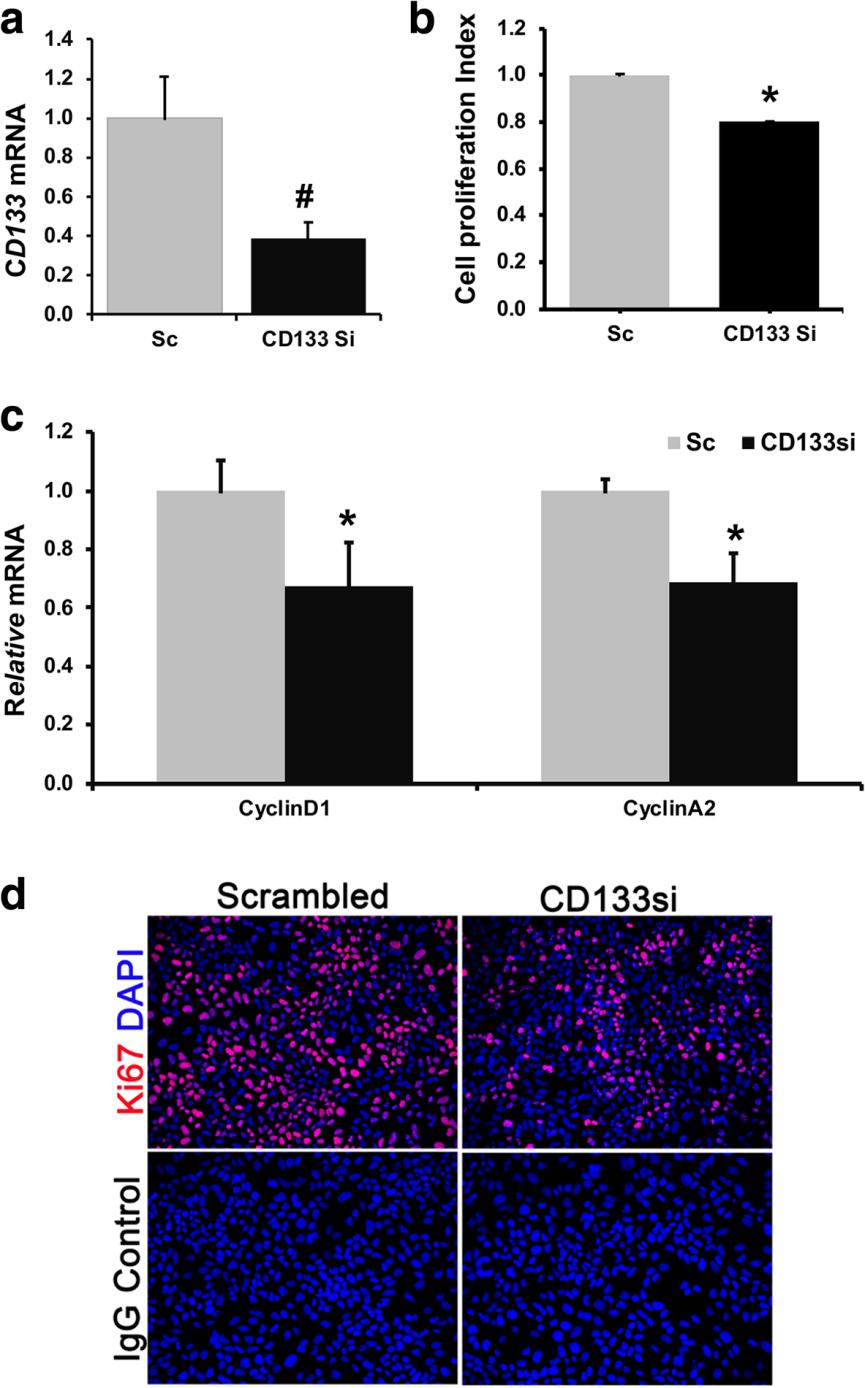
CD133 expression is associated with cell proliferation in TICs **a** siRNA mediated gene silencing of *CD133* for 72 h in TICs. Results represent mean ±S.E from three independent experiments (*n* = 3 # *p* < 0.005). **b** Effect of *CD133* silencing on cell proliferation as determined by BrdU incorporation assay (*n* = 6 *p < 0.05). **c** Effect of *CD133* silencing on the expression level of cell cycle regulatory genes, *CyclinD1* and *CyclinA2.* Expression level was normalized to scrambled siRNA control (Sc) and represented as relative fold, *n* = 3 *p < 0.05. **d**. Immuno-staining for proliferation marker, Ki67 (red) in Scrambled and CD133 silenced TICs. Isotype IgG was used as antibody staining control. Nuclei stained with DAPI (Blue). Scale bar = 25 µm. Images represent four independent experiments

Next, we examined whether CD133 has any regulatory role in the proliferation of TICs. Silencing *CD133* gene expression by 60% reduced cell proliferation by 20% compared to scrambled control (Sc) as determined by BrdU incorporation assay (Fig. 3a, b). In addition, expression level of *CyclinD1* and *CyclinA2* mRNA levels were decreased by 40% in *CD133* silenced cells compared to Sc (Fig. 3c). Immunofluorescence staining further demonstrated a decrease of Ki67 positive cells in *CD133* silenced cells compared to Sc (Fig. 3d).

### CBP-beta-catenin signaling inhibits PTEN and activates AKT-beta catenin pathway in murine liver TICs

**Fig. 4:**
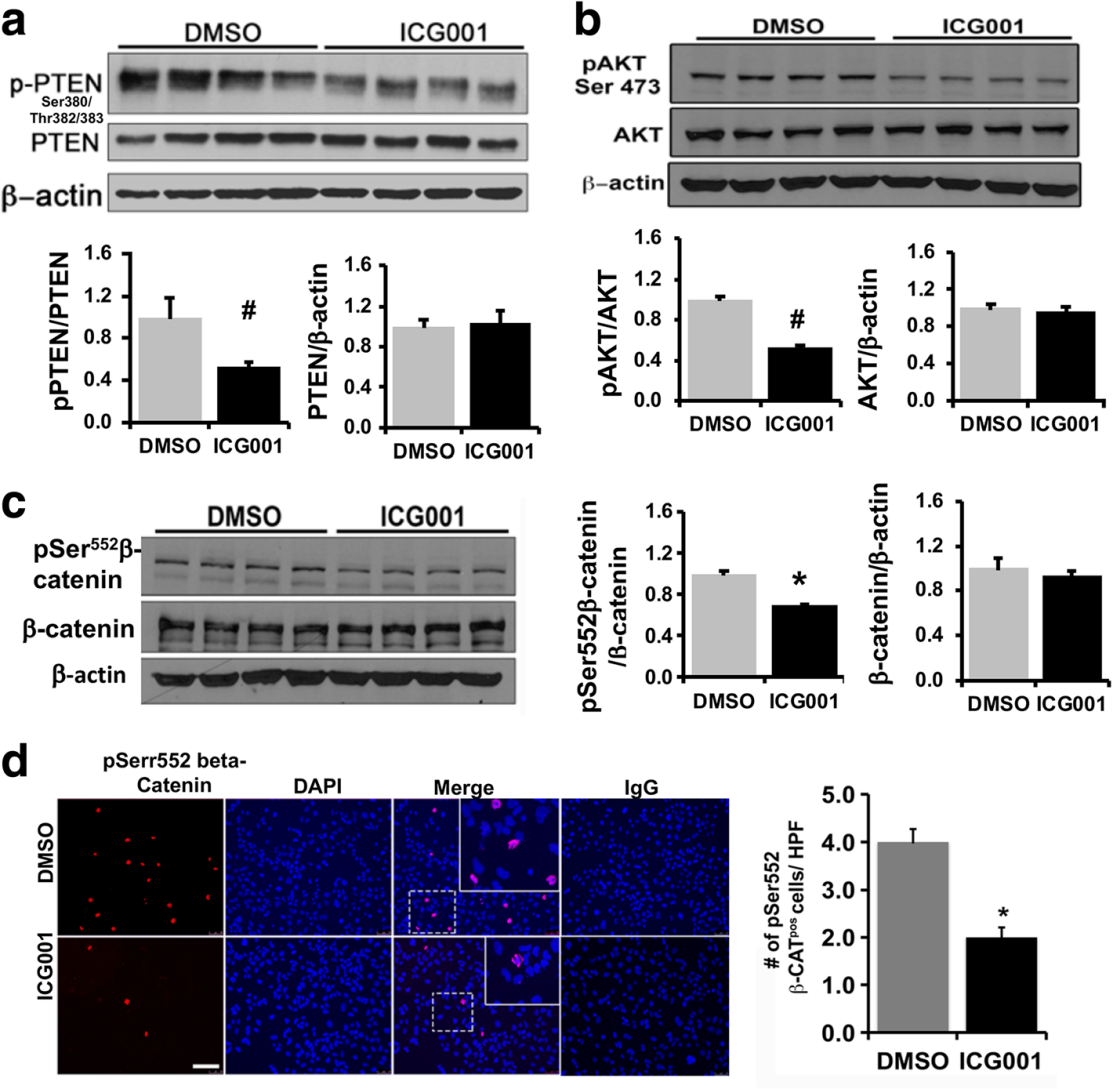
ICG001 treatment activates PTEN and inactivates AKT and beta-catenin in murine TICs*:* **a** Western blot showing PhosphoSer380/Thr382/383-PTEN and total PTEN in ICG001 treated and DMSO control samples. **b** Western blot of PhosphoSer473-AKT and total AKT in ICG001 treated and DMSO controls. Protein band intensity was calculated by densitometry quantification by Image J Software, NIH. Results represent mean ±S.E from four independent experiments. (*n* = 4, #*p* < 0.005). Data was normalized to DMSO controls and represented as relative expression fold. **c** Western blot analysis for pSer552-beta-catenin in ICG001 treated samples and DMSO controls. Data was normalized to DMSO controls and represented as relative expression fold (*n* = 4 *p < 0.05). **d** Immunofluorescence staining for phospho-Ser552-beta-catenin in DMSO and ICG001 treated TICs. Isotype IgG staining was performed as staining control. Number of phospho-Ser552-beta-catenin positive cells per high power field (HPF) was manually counted and compared to DMSO control. Results represent mean ±S.E from four independent experiments (*n* = 5, *p < 0.01). Scale bar = 25 µm

**Fig. 5:**
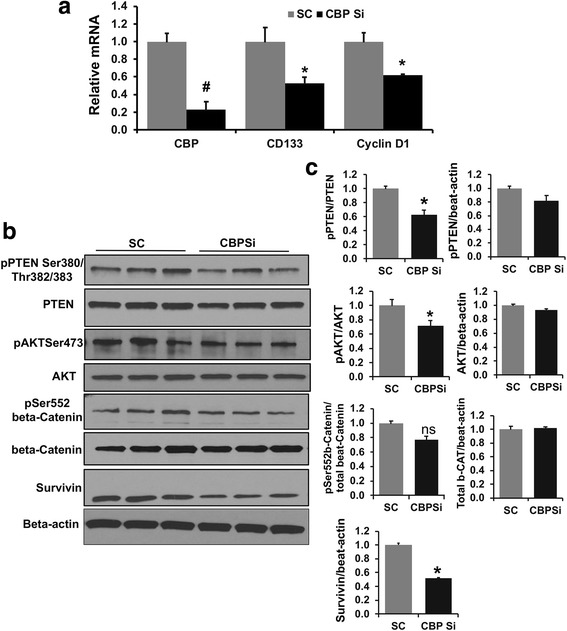
*CBP* silencing downregulates *CD133*, cell proliferative genes and modulates PTEN-AKT pathways in TICs. **a** mRNA expression of *CBP, CD133* and *CyclinD1* in *CBP* silenced TICs. Results represent mean ±S.E from three independent experiments. Data was normalized to scrambled controls (SC) and represented as relative expression fold. (*n* = 3 #*p* < 0.01, *p < 0.05). **b** Western blot analysis to show changes in protein phosphorylation in *CBP* silenced cells. **c** Densitometry quantification of protein band intensity by Image J Software, NIH. Results represent mean ±S.E from three independent experiments. *n* = 3, *p < 0.05

Our previous study has demonstrated that CBP-beta-catenin pathway is highly activated in murine liver TICs and regulated by the AKT pathway [21]. In this study we further extended our efforts to elucidate the functional link between CBP-beta-catenin and AKT signaling in TICs. First, we examined the effect of ICG001 on the upstream AKT modulator and tumor suppressor protein, PTEN. Phosphorylation of PTEN at Ser380/Thr382/383 amino acids is known to regulate PTEN activity and stability negatively [26, 27]. Western blot analysis demonstrated that ICG001 treatment decreased the phosphorylation of PTEN at Ser380/Thr382/383 by 50% compared to DMSO control with no change in total protein levels (Fig. 4a). PTEN inhibition leads to increased phosphorylation of AKT at Ser473, which determines its maximum kinase activity towards its downstream substrates [28, 29]. Western blot analysis demonstrated ~ 50% decrease in the level of phospho-Ser473 AKT by ICG001 compared to DMSO control (Fig. 4b). Additionally, ICG001 decreased the phosphorylation of beta-catenin at Ser552 (Fig. 4c), a known AKT phosphorylation site [30]. Immuno-fluorescence staining showed 50% less phospho-Ser552-beta-catenin positive cells following ICG001 treatment, further confirming the inactivation of AKT by ICG001 (Fig. 4d). To find out whether the ICG001-mediated decrease in CD133 expression and phosphorylation of PTEN and AKT is a directly regulated by CBP, *CBP* was silenced in TICs for 48 h and CD133 expression and phosphorylation of PTEN and AKT was examined. Silencing of *CBP* by 80% in TICs downregulated *CD133* mRNA by 50% compared to scrambled siRNA control (SC) (Fig. 5a). *CBP* silencing caused a 40–50% decrease in CBP target genes *CyclinD1* and Survivin compared to negative control SC (Fig. 5a). Western blot analysis revealed that *CBP* silencing caused a significant 38% decrease in the level of phosphorylated PTEN and a non-significant drop in total PTEN levels (Fig. 5b). *CBP* silencing also caused a 30% decrease in the level of phospho-Ser473 AKT, whereas a non-significant decrease was noticed in the level of phospho-Ser552 beta-catenin (Fig. 5b). In summary, these results demonstrate that CBP-beta-catenin signaling inactivates PTEN which results in the activation AKT pathway in TICs. To determine the specificity of ICG001, western blot was performed to check its effect on the activation of ERK. There was no change observed in the phosphorylation levels of ERK in response to ICG001 (Additional file 3: Figure S1).

### CBP-beta-catenin mediated inactivation of PTEN is PP2A dependent

**Fig. 6:**
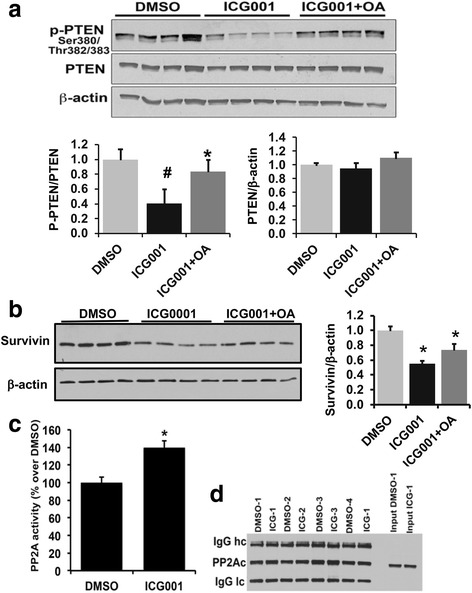
ICG001 mediated activation of PTEN is PP2A dependent **a** Western blot analysis to show the effect of ICG001 and PP2A inhibitor Okadaic acid (OA) on PTEN phosphorylation. Results represent mean ±S.E from four independent experiments. (*p < 0.01, DMSO vs ICG001; #*p* < 0.05, ICG001 vs ICG001 + OA). **b** Western blot analysis showing Survivin expression in ICG001−/+ OA treated samples. Results represent mean ±S.E from four independent experiments. *p < 0.05 (DMSO vs ICG001 and ICG001 vs ICG001 + OA). **c** ICG001 activates PP2A in murine TICs*.* PP2Ac was immuno-pelleted from cell extracts and PP2A activity was measured using PP2A activity assay kit as described in material and methods. Results represent mean ±S.E from four independent experiments

Protein phosphorylation in vivo is a dynamic and highly regulated process. This is mainly achieved by the balanced activities of protein phosphatases and kinases. We hypothesized that PTEN de-phosphorylation in response to ICG001 treatment might be mediated via PP2A. PP2A is one of the serine/threonine phosphatases involved in the regulation of many cellular processes and is an important regulator of canonical WNT signaling. Its activity is inhibited in many cancers and hence is believed to be a tumor suppressor [31, 32]. To find out the molecular mechanism of ICG001 mediated de-phosphorylation of PTEN and potential role of PP2A, ICG001 treatment was performed in the presence of 5 nM okadaic acid, a dose that inactivates PP2A [33]. Our results show that ICG001 mediated decrease in PTEN phosphorylation was reverted upon co-treatment with okadaic acid (Fig. 6a). Okadaic acid itself did not cause any significant change in the phosphorylation of PTEN, suggesting a minimum base line PP2A activity in TICs (Additional file 4: Figure S2). Additionally, okadaic acid co-treatment in the presence of ICG001 was able to increase the levels of Survivin, a known CBP target [19] (Fig. 6b). The findings that PTEN phosphorylation is restored in the presence of okadaic acid prompted us to ask whether PP2A activity is modulated by ICG001. To specifically measure endogenous PP2A activity, PP2Ac was immunoprecipitated from the cell extracts prepared from TICs that were pre-treated with ICG001 or DMSO and PP2A activity was measured as described in the methods section. We found that ICG001 significantly elevated PP2A activity compared to DMSO controls (Fig. 6c). Figure 6d shows western blot analysis to validate PP2AC immunoprecipitation assay. Studies have reported that PP2A activity is regulated in part via phosphorylation or methylation of its catalytic sub unit [34]. ICG001 did not affect either total PP2AC protein levels, or its phosphorylation and methylation levels as shown in Additional file 5 Figure S3.

## Discussion

Better understanding of the signaling pathways that induce stemness and promote the survival of tumor initiating cells is critical for preventing cancer recurrence and drug resistance. The presence of cancer stem cell or tumor initiating cell phenotype has been identified across multiple human cancers including lung, gastric, breast, pancreas and liver [35–39]. Tumor initiating cells are identified by the expression of stem cell markers. CD133 is one of the common cell surface glycoprotein expressed on stem cells and cancer stem cells and has been shown to correlate with drug resistance and cancer recurrence [14, 40, 41]. Cancer patients with high CD133 expression levels were found to have a shorter overall survival and higher recurrence rates than patients with low CD133 expression levels [42, 43]. In experimental models, CD133 positive cancer cells showed higher tumorigenic potential in vivo than CD133 negative cells [37, 44]. Recent studies have shown evidences to support the notion that high WNT activity correlates cancer cells with stem cell capabilities [15, 45]. Therefore, targeting WNT pathway is a clinically relevant therapeutic approach to prevent the development and expansion of cancer stem cells and tumor initiating cells. Even though several WNT inhibitors have been discovered to date, pharmacological targeting of WNT pathway faces many challenges due to highly complex protein-protein interactions, dynamic post-translational modifications and cross talk with other signaling pathways associated with WNT signaling. Therefore, identification of specific cancer stem cell targets within the WNT signaling cascade will greatly help us in understanding the mechanism of tumor cell origin and cancer recurrence.

Using the specific CBP-beta catenin inhibitor ICG001 and in vitro gene silencing methods, we show that *CD133* expression is regulated by CBP-beta-catenin signaling in murine TICs and HCC cells. In TICs, *CD133* expression was positively correlated with cell proliferation and expression of multiple cell cycle regulatory genes. A recent study showed that CD133 interacts with PI3K subunit p85 and activates AKT pathway in glioma cancer stem cells and enhance cell proliferation [46]. Upregulation of CD133 in colorectal cancer cells was associated with activation of Ras-Raf-ERK pathway [47]. CD133 expression is regulated via epigenetic modifications such as demethylation in some cancers [48, 49]. The mechanism by which CBP regulates the expression of CD133 in TICs needs further investigation.

Our previous study has demonstrated a cross talk between AKT and CBP-beta-catenin signaling in TICs that favors cell proliferation and survival [21]. Here we extended our efforts to further elucidate this signaling cross talk in TICs. Our data demonstrates a novel interplay between CBP-beta-catenin and the PP2A-PTEN-AKT-beta-catenin pathway in TICs. CBP-beta catenin signaling inactivates tumor suppressor protein PTEN in TICs, thus providing TICs a survival advantage. Activation of PTEN (less phosphorylated) by ICG001 was further confirmed by the decreased phosphorylation of AKT and beta-catenin (Fig. 4). ICG001 and CBP silencing caused a significant decrease in PTEN phosphorylation. PTEN activity and stability is regulated by its phosphorylation [26, 27]. We noticed a non-significant drop in total PTEN in *CBP* silenced samples. The difference in the fold activation level of PTEN in the presence of ICG001 and *CBP* silencing could  be due to decreased total PTEN protein levels and potentially a time dependent effect.

PP2A is an important regulator of canonical WNT signaling [50]. Its expression is down-regulated in many cancers [31, 32]. We show a novel finding that PP2A activity is induced by ICG001, which resulted in the de-phosphorylation and activation of PTEN. This suggests that CBP-beta catenin signaling act as a negative regulator of PP2A in liver TICs. PP2A-PTEN interaction has been shown to have a regulatory role in prostate cancer progression [51]. Neither PP2AC protein levels or its phosphorylation and methylation levels changed following ICG001 treatment. This demonstrates that ICG001-mediated increase in PP2A activity is not regulated at the catalytic subunit level. A recent study by Sangodkar et al. reported that activation of PP2A by small molecule activator SMAP, inhibited lung cancer cell growth in xenograft models [52]. Authors also demonstrated that SMAP activates PP2A by binding to its Aα regulatory subunit of PP2A that causes conformational changes and PP2A enzyme activation [52]. In summary our data show that CBP-beta-catenin signaling induces stemness to tumor initiating cells, promotes cell proliferation, and inhibits PP2A-PTEN pathways in liver TICs.

## Conclusions

Targeting a specific signaling pathway that induces stemness to tumor initiating cells is critical for developing novel therapies to prevent cancer initiation and recurrence. Our study demonstrates a regulatory role for CBP-beta catenin signaling in inducing stem cell phenotype and highlights a novel mechanistic link between CBP-beta-catenin and the tumor suppressor proteins PP2A-PTEN in liver TICs. A deeper understanding of cancer stem cell biology will facilitate in elucidating the mechanisms of tumor cell origin and resistance to targeted therapy.

## Additional files

Additional file 1: **Table S1.** List of primary antibodies. (ZIP 125 kb)

Additional file 2: **Table S2.** List of qPCR probes. (ZIP 50 kb)

Additional file 3: **Figure S1.** Effect of ICG001 on activation of ERK1/2. Western blot analysis for phospho-ERK and total ERK in DMSO and ICG001 treated samples. Protein levels were normalized to beta-actin and ratio of pERK1/2 to total ERK1/2 was calculated to determine the level of activation. Results represent four independent experiments. Densitometry quantification of protein intensity was performed by Image J Software, NIH. (ZIP 319 kb)

Additional file 4: **Figure S2.** PP2A is induced by ICG001 in TICs. Western blot analysis shows the effect of ICG001 and OA alone or in combination on PTEN phosphorylation. Densitometry quantification of protein intensity was performed by Image J Software, NIH. *N* = 3, *p < 0.05. (ZIP 366 kb)

Additional file 5: **Figure S3.** ICG001 mediated PP2A activation is independent of posttranslational modification of PP2Ac. Western blot analysis for phospho-PP2Ac and methyl PP2Ac in DMSO and ICG001 treated samples. Results represent four independent experiments. (ZIP 524 kb)
