# Supplementary figures and images for: Inhibition of CREB binding protein-beta-catenin signaling down regulates CD133 expression and activates PP2A-PTEN signaling in tumor initiating liver cancer cells
Source: Cell Commun Signal. 2018 Mar 12;16:9. doi: 10.1186/s12964-018-0222-5 (PMC5848530; doi:10.1186/s12964-018-0222-5)

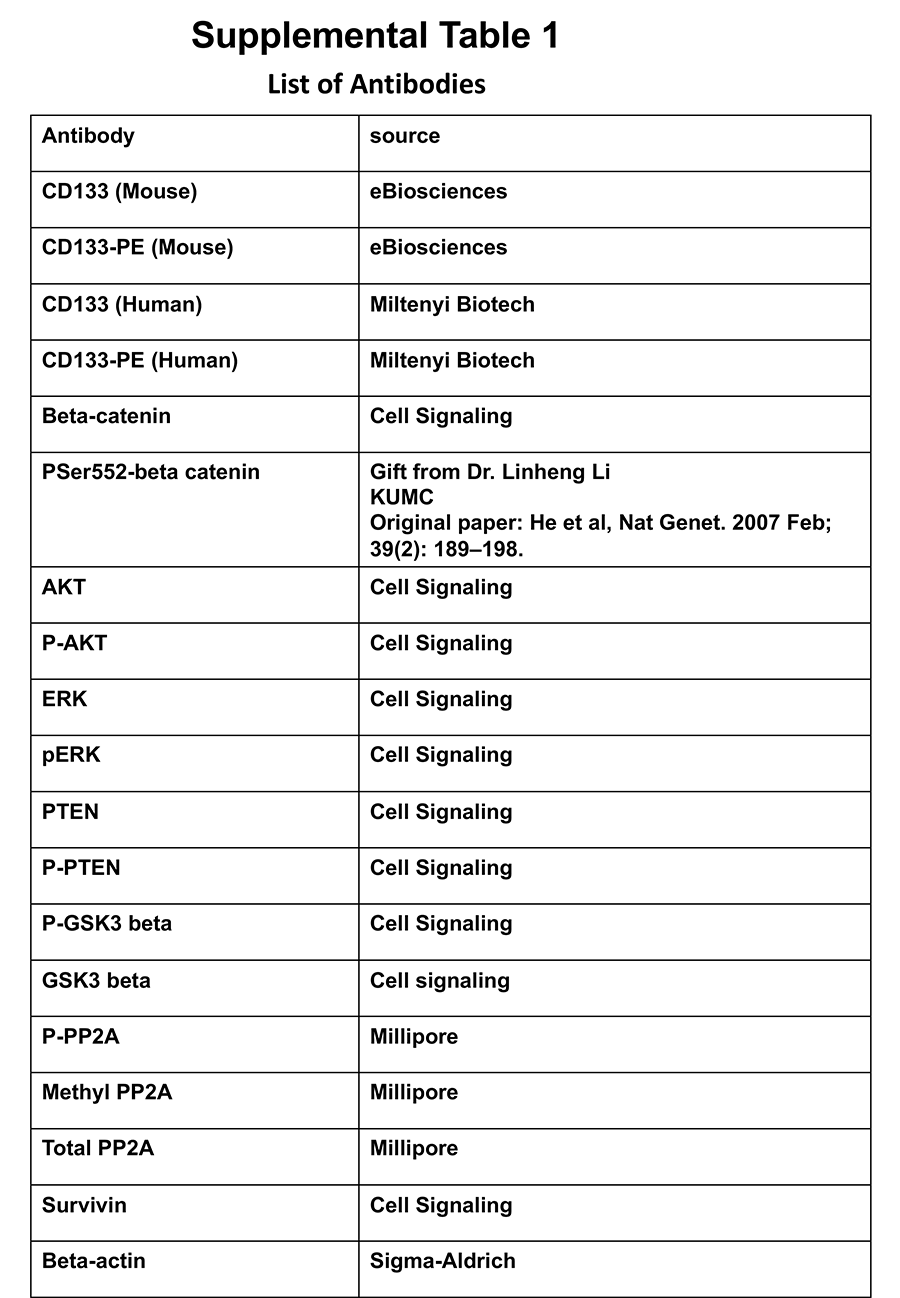

Supplement: Supplementary file 1 — Table S1. List of primary antibodies. (ZIP 125 kb) [file 12964_2018_222_MOESM1_ESM.zip › Supplemental Table1]

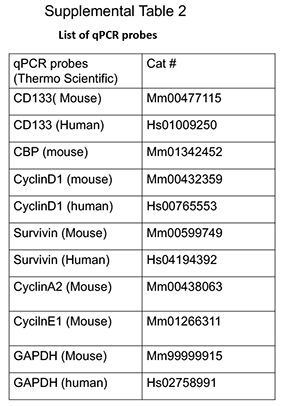

Supplement: Supplementary file 2 — Table S2. List of qPCR probes. (ZIP 50 kb) [file 12964_2018_222_MOESM2_ESM.zip › Supplemental Table2]

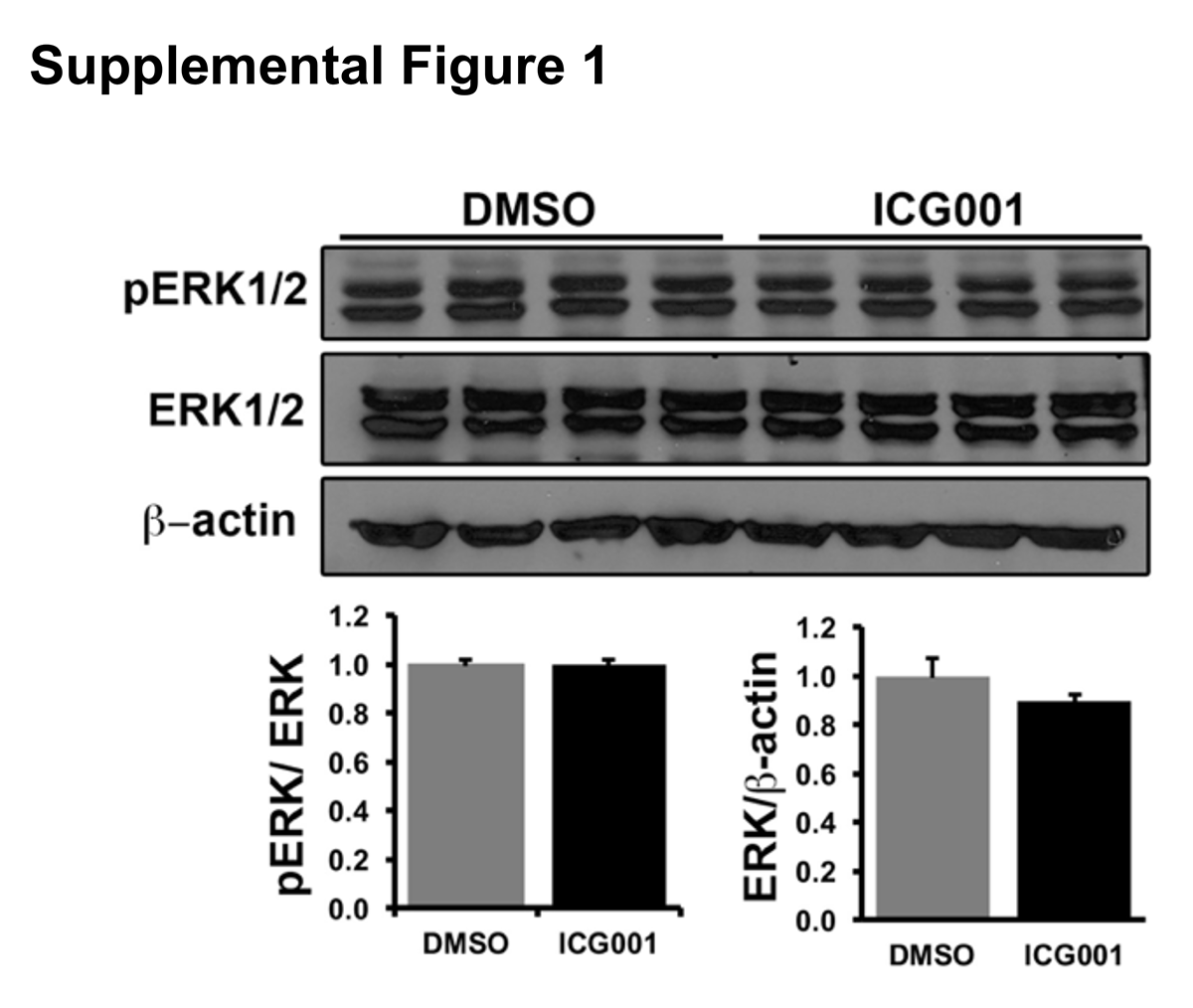

Supplement: Supplementary file 3 — Figure S1. Effect of ICG001 on activation of ERK1/2. Western blot analysis for phospho-ERK and total ERK in DMSO and ICG001 treated samples. Protein levels were normalized to beta-actin and ratio of pERK1/2 to total ERK1/2 was calculated to determine the level of activation. Results represent four independent experiments. Densitometry quantification of protein intensity was performed by Image J Software, NIH. (ZIP 319 kb) [file 12964_2018_222_MOESM3_ESM.zip › Supplemental Figure1]

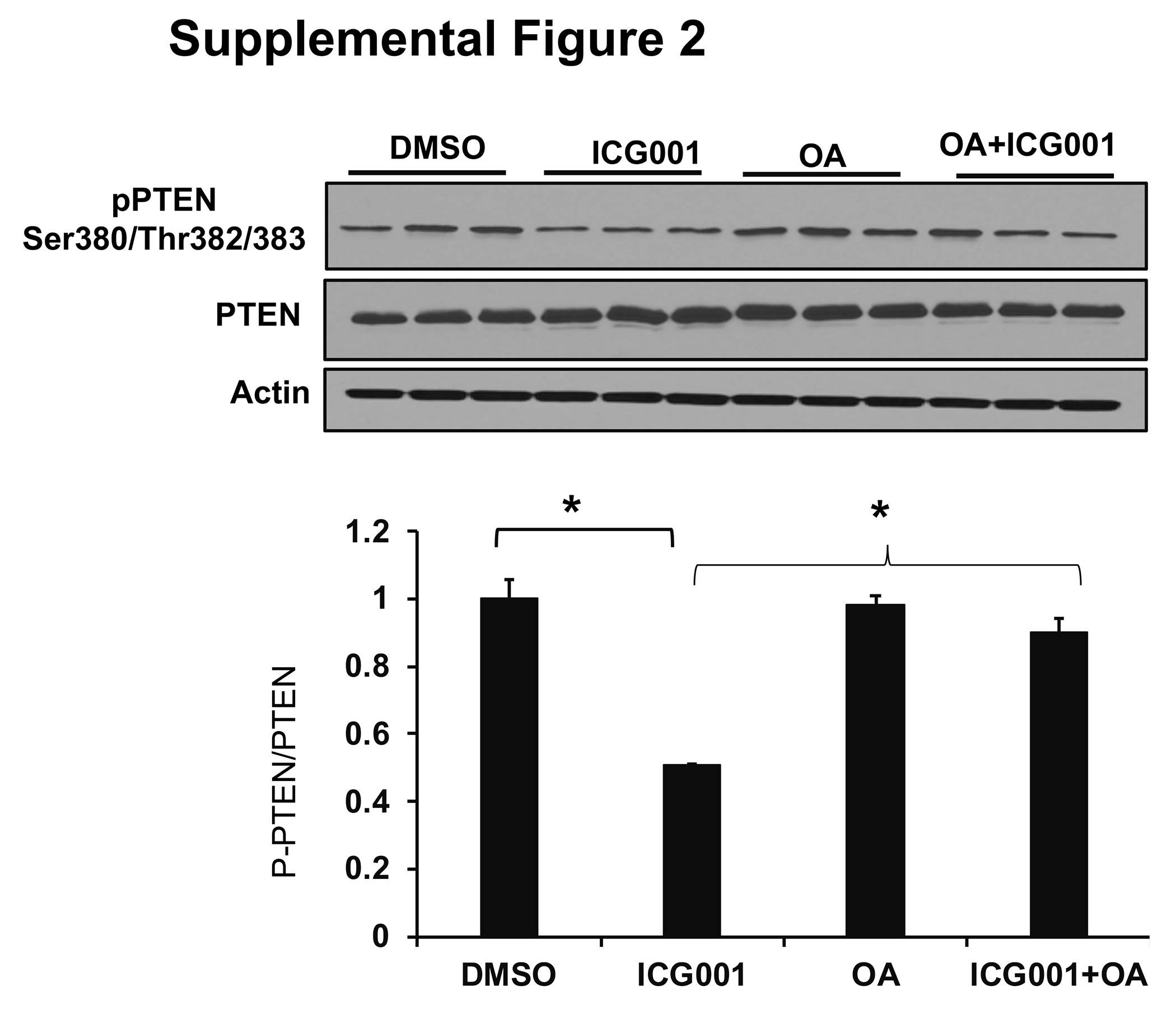

Supplement: Supplementary file 4 — Figure S2. PP2A is induced by ICG001 in TICs. Western blot analysis shows the effect of ICG001 and OA alone or in combination on PTEN phosphorylation. Densitometry quantification of protein intensity was performed by Image J Software, NIH. N = 3, *p < 0.05. (ZIP 366 kb) [file 12964_2018_222_MOESM4_ESM.zip › Supplemental Figure2]

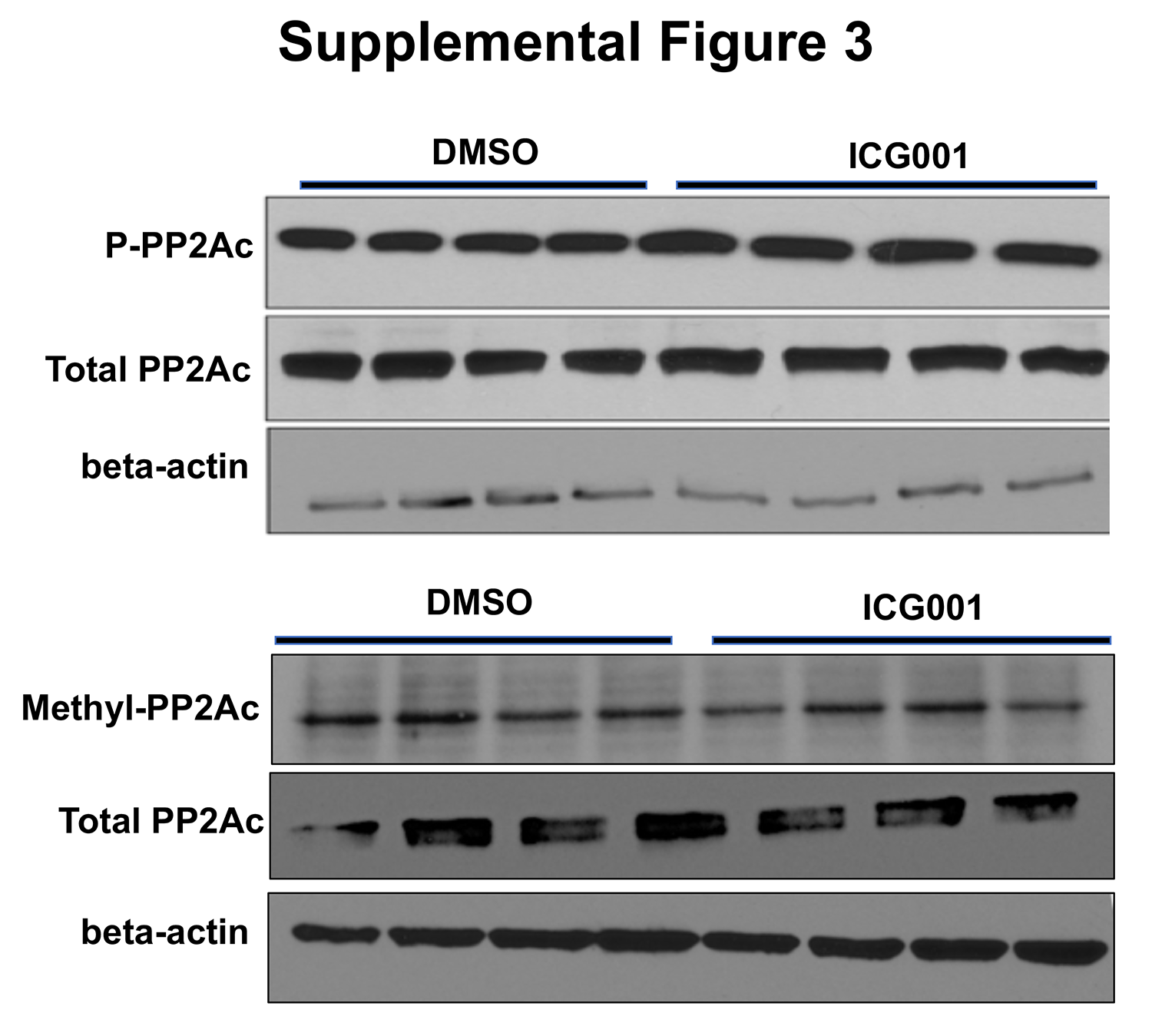

Supplement: Supplementary file 5 — Figure S3. ICG001 mediated PP2A activation is independent of posttranslational modification of PP2Ac. Western blot analysis for phospho-PP2Ac and methyl PP2Ac in DMSO and ICG001 treated samples. Results represent four independent experiments. (ZIP 524 kb) [file 12964_2018_222_MOESM5_ESM.zip › Supplemental Figure3]
